# Barriers to primary care responsiveness to poverty as a risk factor for health

**DOI:** 10.1186/1471-2296-12-62

**Published:** 2011-06-29

**Authors:** Gary Bloch, Linda Rozmovits, Broden Giambrone

**Affiliations:** 1 Department of Family and Community Medicine, St. Michael's Hospital, 80 Bond St., Toronto, Ontario, M5B 1X2, Canada; 2 Department of Family and Community Medicine, Faculty of Medicine, University of Toronto, Toronto, Ontario, Canada; 3 Toronto, Ontario, Canada; 4 Dalla Lana School of Public Health, University of Toronto, Toronto, Ontario, Canada

## Abstract

**Background:**

Poverty is widely recognized as a major determinant of poor health, and this link has been extensively studied and verified. Despite the strong evidentiary link, little work has been done to determine what primary care health providers can do to address their patients' income as a risk to their health. This qualitative study explores the barriers to primary care responsiveness to poverty as a health issue in a well-resourced jurisdiction with near-universal health care insurance coverage.

**Methods:**

One to one interviews were conducted with twelve experts on poverty and health in primary care in Ontario, Canada. Participants included family physicians, specialist physicians, nurse practitioners, community workers, advocates, policy experts and researchers. The interviews were analysed for anticipated and emergent themes.

**Results:**

This study reveals provider- and patient-centred structural, attitudinal, and knowledge-based barriers to addressing poverty as a risk to health. While many of its findings reinforce previous work in this area, this study's findings point to a number of areas front line primary care providers could target to address their patients' poverty. These include a lack of provider understanding of the lived reality of poverty, leading to a failure to collect adequate data about patients' social circumstances, and to the development of inappropriate care plans. Participants also pointed to prejudicial attitudes among providers, a failure of primary care disciplines to incorporate approaches to poverty as a standard of care, and a lack of knowledge of concrete steps providers can take to address patients' poverty.

**Conclusions:**

While this study reinforces, in a well-resourced jurisdiction such as Ontario, the previously reported existence of significant barriers to addressing income as a health issue within primary care, the findings point to the possibility of front line primary care providers taking direct steps to address the health risks posed by poverty. The consistent direction and replicability of these findings point to a refocusing of the research agenda toward an examination of interventions to decrease the health impacts of poverty.

## Background

Poverty is widely recognized as a major determinant of poor health [1-3]. The powerful link between income and health has been well documented -- people living on low income consistently have higher rates of morbidity and mortality due to chronic and acute illnesses [4-7]. This impact is particularly worrisome amongst children, who exhibit a higher risk of detrimental health outcomes throughout their life-course regardless of later socioeconomic status [8]. Nonetheless, there are few studies of how family physicians respond to the social problems, such as poverty, inadequate housing, or food insecurity, experienced by their patients [9]. This gap may partly be due to the fact that research into the social determinants of health has largely been focused on policy-level and public health-based interventions. Very little has been done to directly examine primary health care providers' responsiveness to income as a risk factor for health. Moreover, while family medicine has a strong history of addressing issues once considered social, such as smoking and obesity, income remains largely unaddressed in primary care. As a step towards the development of potential direct interventions by primary care providers, this study examines some of the current barriers to effectively addressing poverty as a risk to health in the province of Ontario, Canada.

There is a nuanced literature discussing access to health care for different populations [10], however access to care for people who live at low income has been less well-explored. Existing evidence points to significant barriers to people living on low income receiving high quality primary care that is responsive to their social circumstances. There have been reports of inequity in access to primary care physicians in Canada based on income [11], although this may be less than previously thought [12]. People living in poverty also face a range of practical barriers to accessing primary care. For example, lack of access to transportation [13], not having a valid health insurance card [14,15], and difficulty making and keeping appointments are commonly cited [16]. Inflexible practice rules and billing structures that make it disadvantageous for family physicians to serve patients with complex care needs have also been identified [17].

Beyond barriers to access, the quality of interactions between family physicians and people living in poverty is more complex than simple utilization rates suggest. When patients living in poverty access health services, they are more likely to have shorter consultation times than their wealthier peers [18], and are less likely to be involved in treatment decisions [19]. Moreover, despite their complex care needs, low-income patients may be reluctant to disclose social problems due to stigma and/or discrimination while family physicians may be reluctant or feel ill-equipped to probe for these issues [20]. The resulting lack of knowledge of patients' circumstances can lead to treatment plans patients are unable to follow [21]. Family physicians may also lack knowledge of local social and community resources that could benefit their low-income patients [22]. Unwelcoming attitudes or disrespect towards low-income patients and discrimination by family physicians based on ethnicity, immigration status, and gender, in conjunction with low income, may also constitute a barrier to care [23,24]. The effects of these barriers may be compounded by patients' shame at their personal circumstances, desire to be self-reliant, and real or perceived feelings of discrimination in the health care system [25].

This study is part of a larger program of research that aims to support the development of evidence-based educational curricula and practice-oriented tools for family physicians. The aim of this study is to explore expert informants' perspectives on the barriers to primary care responsiveness to poverty as a health issue. As a study solely of expert informants, this is intended as both a test of the relevance, in a highly resourced jurisdiction with near-universal health care insurance such as Ontario, of what the literature has previously demonstrated in other contexts, and as a stepping stone to a more comprehensive exploration of primary care providers' approaches to poverty, through a larger study of a broad group of primary care providers and people who live in poverty.

## Methods

This qualitative pilot study employed semi-structured, in-depth interviews with expert informants in the province of Ontario, Canada, to develop a provisional map of issues pertinent to optimizing primary care provision for low-income patients. This method is well-suited to the exploration of complex social phenomena such as interactions between physicians and patients, as well as barriers and enablers to care provision for complex-needs populations. Ethics approval for this study was obtained from the St. Michael's Hospital Research Ethics Board.

### Participants

A list of fourteen key informants with established academic and/or frontline expertise in the social determinants of health, including income, housing and food security in relation to primary care was compiled by the principal investigator Invitations to take part in an interview were issued by e-mail and participants were informed that the interviews constituted a pilot study intended to support the development of educational curricula for family physicians and other health care providers, and to inform subsequent research with a broad population of family physicians and people living in poverty in Ontario. Two invitees declined to participate due to time constraints.

Purposive (non-probability) sampling, which seeks to maximize theoretical return by allowing for variation within a focused field of inquiry, was used [26]. Participants thus included 2 urban and 1 rural-based generalist physicians, 1 psychiatrist, 1 internist, 1 street nurse, 1 nurse practitioner, and 5 community activists and social policy experts representing community-based organizations, street outreach programs, hospital-based programs in Toronto, and Toronto-based research hubs including the Centre for Research on Inner City Health, the Wellesley Institute, and the Metcalf Foundation. The sample was not based on conventional family physicians as many patients living in poverty have more contact with advocacy groups, street outreach programs and hospital-based physicians than they do with mainstream primary care. Participants included 8 male and 4 female respondents, all but two of whom have focused on the needs of low income patients for much or all of their careers.

### The Interview Process

Nine face-to-face and three telephone interviews lasting an average of 52 minutes (range 40-75 minutes) were conducted. A semi-structured interview guide was developed based on issues drawn from the literature reviewed. Interviews were conducted by an experienced, senior qualitative research practitioner and solicited participants' views on: challenges of access and delivery of primary care to low-income patients; family physician awareness of and responsiveness to, income level in relation to health; and interventions at both the level of direct patient care, and at the more broadly socio-political level that might improve access and care for low-income patients. All interviews were digitally audio-recorded for verbatim transcription.

### Analysis

Transcripts were checked against sound files for accuracy, entered into HyperResearch software for qualitative data analysis and coded for both anticipated and emergent themes. A coding framework was developed in discussion with the study team. Independent coding of a selection of transcripts by another study team member was used to ensure consistency in application of analytic codes.

For the analysis, the method of constant comparison was used and included searches for disconfirming evidence. As this was only a pilot study, no respondent validation exercise was conducted. It is anticipated that respondent validation will be included in subsequent phases of the larger research program of which this study was a part.

## Results

The study revealed a wide range of patient- and provider-centred barriers to low-income patients accessing care which is responsive to their poverty as a risk factor for health. These included structural, attitudinal and, for providers, knowledge-based barriers. On the patient side, issues that replicate findings reported in the existing literature included lack of access to transportation in both urban and rural settings, not having a valid health insurance card and difficulty making and keeping appointments. Also echoing established findings were limited help-seeking linked to stigma and shame at personal circumstances, low literacy levels, substance abuse issues and cognitive impairment. On the provider side, inflexible practice rules that are difficult for low-income patients to comply with, billing structures that discourage longer appointments, unwelcoming practice environments and a lack of familiarity with the social security system and relevant community-based resources were frequently named as contributing to sub-optimal care for this patient group. Beyond these familiar findings, however, additional evidence emerged that indicates the need for intervention at the level of direct physician/patient interaction rather than at the policy or population level.

### Failure to respond to the social determinants of health as a lived reality

Several participants argued strongly that while the average family physician in Ontario would likely be familiar with the social determinants of health as a concept, few would have a substantive appreciation of the lived reality of social disadvantage:

They might see it ... in terms of a theoretical academic construct but I don't think they'd actually understand the real reality of it in terms of what it actually means for a person to get × amount of dollars and be forced to try to live on those dollars ... [PH01]

This was not presented as evidence of ill-will or a lack of compassion but as a consequence of the social distance between physicians and their low-income patients:

... people that go to university and become health professionals tend to be people that are from a higher social class ... and once you get working as a health professional that makes good money you also continue to occupy a social class where ... you can't even imagine that people are hungry and you can't even imagine that people can't afford healthy food or have to eat Kraft Dinner every day ... it's just so outside your lived experience that you don't think about it. [PH03]

A concomitant gap was the physicians' lack of awareness of patients' medically relevant personal details. For, although income level is widely acknowledged to be a powerful determinant of health, many participants felt that physicians generally did not have the demographic data necessary to identify and respond to the risks associated with low income in the way they would routinely respond to health risks such as smoking or hypertension:

I suspect that most physicians would be reluctant or it would not be in their consciousness to enquire about people's economic circumstances, employment, income, debt, nature of their housing ... There's not a high degree of awareness about it ... If they bring in a general Welfare form or an ODSP application or an unemployment form, obviously that should alert the physician the person's not exactly well off. I'm not sure how much would be pursued thereafter ... it's not in people's consciousness to look, to gaze beyond the individual patient and try to see what the circumstances of their life are that might be making them sick ... [PH12]

Lack of awareness of patients' social circumstances was also cause for concern because of the likelihood that care plans devised in the absence of this information would be difficult or impossible for patients to follow:

... you cannot treat cellulitis unless you know somebody's social circumstances and are they able to take the medication QID four times a day, are they able to afford the medication, do they have a watch ... can they eat ... do they have enough to eat? ... [PH07]

... people all the time ... say to me, "I'm supposed to be on these four medications. Which one is the most important because I can only afford one?" [PH03]

This, in turn, might lead to medications being prescribed in a futile manner or as a substitute for more time-consuming but ultimately more appropriate care:

You might live in a mould-infested basement that's really the cause of your respiratory illness and the physician might never think to say, "Where are you staying and what is it like there?"... maybe he spends a year giving you medicines that don't work because the cause of your problem is your crappy housing. [PH03]

Care plans devised in the absence of adequate information about patients' personal circumstances were also seen as contributing to negative attitudes toward low-income patients since their inability to follow plans as indicated could easily be interpreted as non-compliance:

... a physician says "... take this antibiotic three times ... on a full stomach," and I always laugh hysterically and the women who I know who are working poor laugh because they know that, "Yeah, three meals, like what's he talking about, three meals? A full stomach!" [PH11]

... I had ... an old guy that needed diabetes medicine who lived in a shelter in Toronto ... he was elderly and he had mobility issues and he didn't take any of his diabetic medication because the side-effect that it caused for him was diarrhoea and he was living in a shelter with 60 younger men and two toilets ... he had no chance of getting to the toilet if he needed to quickly so he wasn't going to take his pills ... because he couldn't manage in that environment. [PH03]

### Prejudicial attitudes and feeling overwhelmed at the scope of the problem

All participants believed that prejudicial attitudes on the part of family physicians were a considerable barrier to low-income patients accessing optimal care. Many felt that physicians often shared in the commonly-held belief that poor people are poor as a result of their own personal failings, the implication being that they are, on some level, less deserving of attention and healthcare resources than wealthier patients:

I think physicians do recognize that poverty and housing are determinants of health but I think that many of them view ... poverty as a result of individual failings and that poor people essentially bring it on themselves ... most physicians are very hard-working people who ... because they've achieved success through hard work tend to view those who didn't achieve success as people who didn't work hard. [PH08]

... there's a terrible pathology that's applied to people who are homeless that somehow they're the authors of their own misfortune, that they are either bad or weak and that these kinds of factors explain their poverty and their homelessness. [PH09]

... there's more of a judgement ... they smoke too much, they drink too much ... that's where the focus is ... [PH04]

Participants also maintained that many physicians feel that responding to issues such as inadequate income or housing falls outside the duty of care of family physicians, that these are problems that they are not obliged to address and which are, in any case, beyond their reach:

... when I work with homeless patients, I'm mostly focusing on their medical problems, not on how can I solve their homelessness or how can I address their poverty ... I can only imagine that that's probably true for most physicians. [PH08]

Moreover, in contrast with the medical response to other health risk factors such as smoking or hypertension, physicians often have no idea what steps they could take to address health issues related to poverty:

... people have this vague understanding that the relationship between income and illness is a linear one and the higher your income the lower your health risks ... but nobody really equips us to translate that. We know that hypertension is a health risk and we know that we can order anti-hypertensives ... so it's really easy for me to intervene on that issue because I know what to do ... and it takes, like one second to write a prescription ... if ... people's shitty housing or lack of housing causes their health problems, I don't know what I can do with that ... I don't see that as something that medicine can deal with. I see that as something that the government maybe needs to deal with, or ... some social worker or something. [PH03]

## Discussion

This study confirms the relevance to a jurisdiction such as Ontario of the findings of previous studies that have looked at addressing the primary care needs of patients who live in poverty, and adds to their findings. Given the numerous barriers identified, these results place renewed emphasis on the need for primary health care providers to consider and address income as a distinct risk to health, and for researchers to explore these issues with a broader group of primary care providers and people who live in poverty.

The context of this study is significant. Ontario is a well-financed jurisdiction with a strong, publicly managed and financed health system. Nevertheless, the barriers to addressing the health risks posed by poverty are similar to those identified in previous studies conducted in other settings. These similarities may point to structural deficiencies in primary care practice that repeat across jurisdictions.

Poverty as a social determinant of health has traditionally been tackled by public health practitioners and public policy developers. However, the barriers identified here are, in many cases, concrete and surmountable and point to the potential ability of individual primary care providers to directly mitigate the effect of poverty as a risk to health by means of enhanced education and alteration of routine practices. This may allow for significant action on poverty as a health risk, while simultaneously working toward higher level systemic changes to reduce the impact of poverty on health.

### Limitations of the Study and indications for further research

Despite differences of degree, all participants in this study broadly shared a left of centre political perspective and were already professionally focused on the social determinants of health. As a result, changes such as the re-structuring of physician remuneration, and the need for more flexibility in the organization of primary care facilities, are consistently supported. The views of healthcare providers situated elsewhere along the political spectrum and who do not have the social determinants of health as a primary focus may diverge from those expressed in the study. Second, all of the participants are based in southern Ontario, and most in Toronto. Efforts should be made in further research to hear voices from throughout Ontario, to ensure the adequate incorporation of views from more remote, rural and smaller urban areas. Third, while expert informants shared their views of what they believe typified interactions between family physicians and their low-income patients, further research is necessary to directly elicit the views and experiences of a range of family physicians and low-income patients. Finally, because of their involvement in advocacy work or the provision of healthcare to extremely marginalized populations, many of these participants focused on the most extreme end of the poverty spectrum. As a result, the working poor and other less visibly marginalized populations were inadequately represented in this study.

## Conclusion

This study confirms the relevance, to a highly resourced jurisdiction with near-universal health care insurance such as Ontario, of the findings of previous studies that have found significant barriers exist to low-income patients obtaining high quality primary care, and to primary care practitioners addressing their patients' poverty as a risk to their health. These barriers are now well known and well replicated. The consistency of these findings suggests it may be time to shift the research and clinical discourse away from identification of barriers and toward an examination of primary care-based interventions into poverty as a risk to patients' health.

## Competing interests

GB is actively involved in education and advocacy work focused on educating primary care providers, policy makers, and the general public about the health impacts of poverty. He is a founding member of the advocacy group Health Providers Against Poverty, and Chair of the Ontario College of Family Physicians' Committee on Poverty and Health.

## Authors' contributions

GB conceived of the research question and oversees the broader research program, secured funding for the project, assisted in the development of interview guides and analytic frameworks, and was involved in every stage of the writing of the manuscript. LR guided the development of the qualitative research protocol, conducted all interviews and analysis, and was involved in every stage of the writing of the manuscript. BG conducted the literature reviews for the project, participated in conceptual meetings concerning the broader research program, commented on the analytic framework, and edited drafts of the manuscript. All of the authors reviewed and approved the final manuscript.

## Authors' information

GB is a family physician with an inner city practice at St. Michael's Hospital in Toronto. He is Assistant Professor in the Faculty of Medicine at the University of Toronto. He is Chair of the Ontario College of Family Physicians' Committee on Poverty and Health, and founding Primary Care Director of Inner City Health Associates, a group of over sixty physicians working with the homeless in Toronto. He frequently educates health providers and trainees about interventions into poverty as a health issue. He is regularly called on by the media to discuss issues related to poverty and health.

LR is an independent qualitative health research consultant in Toronto. She holds a DPhil from Sussex University. Over the past ten years she has worked with research teams at many leading teaching hospitals and academic research centres in the UK and Canada. In addition she has worked with many governmental and quasi-governmental agencies, health and social care non-profit organizations and grassroots community groups.

BG holds a Masters in Health Science in Health Promotion from the University of Toronto. He has a strong research interest in the social determinants of health, and a long history of organizing and advocacy on issues related to social justice and health. He is currently director of the Transgender Equality Network Ireland.

## Pre-publication history

The pre-publication history for this paper can be accessed here:

http://www.biomedcentral.com/1471-2296/12/62/prepub
